# An emerging prognosis prediction model for multiple myeloma: Hypoxia-immune related microenvironmental gene signature

**DOI:** 10.3389/fonc.2022.992387

**Published:** 2022-08-30

**Authors:** Zhengyu Yu, Bingquan Qiu, Linfeng Li, Jing Xu, Hui Zhou, Ting Niu

**Affiliations:** ^1^ Department of Hematology, West China Hospital, Sichuan University, Chengdu, China; ^2^ Department of Biochemistry and Molecular Biology, School of Basic Medical Sciences, Peking University Health Science Center, Beijing, China

**Keywords:** multiple myeloma, prognosis, hypoxia, immune, tumor microenvironment, immunotherapy

## Abstract

Multiple myeloma (MM), a hematologic malignancy, is characterized by malignant plasma cells clonal proliferation. Many evidences indicated the indirect interaction between hypoxic environment and immune state in MM tumorigenesis, but the underlying mechanism remains unclear. MM-related datasets were downloaded from the Gene Expression Omnibus (GEO) database. The R packages were applied for screening protective differentially expressed genes (DEGs) and risk DEGs. The signature was constructed based the most prognostic gene signature in the training and assessed in the validation cohorts. The immune cell infiltration, the expression of the HLA family and immune checkpoint genes inside the low- and high-risk groups were compared to determine the differences in immune infiltration and immunotherapy responses. Moreover, the expression of HLA families and immune checkpoints inside the low- and high-risk groups was markedly disordered. The results indicated hypoxia- and immune-related genes, including *CHRDL1*, *DDIT4*, *DNTT*, *FAM133A*, *MYB*, *PRR15*, *QTRT1*, and *ZNF275*, were identified and used to construct a prognostic signature. Role of DDIT4 in multiple myeloma was confirmed *in vivo* and *in vitro*. DDIT4 knockdown inhibited MM cell viability, migration and invasion potential as well as promoted myeloma cells apoptosis under hypoxia. Taken together, our study may contribute to the treatment and prognosis prediction of MM.

## Introduction

Multiple myeloma (Da ViàVerify that all the equations and special characters are displayed correctly. etal. (1) is a hematologic malignancy (2). With the emergence of immunotherapies, the therapeutic effect of MM has increased considerably (3, 4). Nevertheless, MM patients still have disease recurrence and aggravation, especially those at high risk (5). At present, there is no recognized biomarkers to stratify the risk and prognosis of MM, which will guide the personalization and timely follow-up treatment of MM patients. Therefore, investigating novel therapeutic targets and disease indicators is essential for MM patients.

Hypoxia remodels tumor-associated environment and drives types of tumor invasions (6). Tumors become more aggressive and metastatic in hypoxic TMEs (7). The characteristics related to hypoxia exhibited predictive potential across cancers (Q. 8). Studies have shown that hypoxia plays special role in MM biology and provides new possibilities for therapeutic strategies (9). Exposure to hypoxia can significantly enhance MM cells viability by inducing angiogenesis, anti-apoptotic effect and etc (10).

Immune status is the ability of the body to exhibit an immune response or defense against pathogens, diseases or foreign substances. Several studies have demonstrated that some immunocytes in the tumor sites of MM patients manifest as immune-senescence and exhaustion (11, 12). The proportion of immature B cells in MRD positively correlates with recurrence (11). Similar to the results observed in chemotherapy, MM underwent evade immunotherapy that associated with rather instability and disordered bone marrow microenvironment (BMME) (13). However, exact roles of interaction between hypoxia and immune state remain ambiguous but promotes the development and occurrence of MM.

Here, we uncovered that combined markers of hypoxia genes and immune genes to provide better prognostic value for patients with MM. We divided 867 samples into high- risk and low- risk groups by consensus clustering and draw K-M survival plot, which revealed that the survival of MM correlated with the hypoxia and immune status of the TME. Next, we revealed that the expression of HLA families and immune checkpoints was markedly disordered between the high- and low-risk groups. The risk score and independent prognosis of each clinical factor were investigated by univariate and multivariate regression. Finally, we identified 8 biomarker signatures by a series of systematic analyses aimed at improving the prognostication of MM. In this study, the prognosis and stratification of MM were evaluated by transcriptomics and microenvironmental characteristics, which provide a new direction for the diagnosis and treatment of MM in the future.

## Materials and methods

### Data source

MM-related datasets (GSE136324, GSE47552 and GSE136337) were downloaded from the Gene Expression Omnibus (GEO) database (https://www.ncbi.nlm.nih.gov/). The GSE136324 dataset, containing 867 MM samples with survival information, was used as a training cohort. The GSE47552 dataset includes 41 MMs and 5 normal samples. The GSE136337 dataset was employed as the external validation set, which included 426 MM samples with survival information. Moreover, the expression matrix of HRGs was constructed based on 200 HRGs downloaded *via* the Molecular Signatures Database (MSigDB) (http://www.gsea-msigdb.org).

### Consensus clustering

“ConsensusClusterPlus” was applied to conduct consistent clustering analysis based on 867 MM samples in the GSE136324 dataset, and the cumulative distribution function (CDF) was employed to evaluate the optimal number of clusters. Moreover, principal component analysis (PCA) employed to verify the results of consistent clustering.

### Screening and functional analysis of differentially expressed (DE-H-IRGs)

First, K-M survival analysis was performed on patients with different hypoxia subtypes using the R package “Survival”, and clusters with the worst prognosis were defined as the group with the high hypoxia status (Hypoxia.high (HH)). Clusters with better prognosis were combined and defined as the group with the low hypoxia status (Hypoxia.low (HL)). The “Limm” package was utilized to screen differentially expressed hypoxia -related genes (DE-HRGs) between the lowest and HH groups (14).

Second, the ESTIMATE was performed to calculate the immune score of MM. The “Maxstat” was subsequently utilized to demonstrate the optimal truncation value of the immune score. Then, MMs were grouped into high and low immune score subgroups with the optimal truncation immune score value. The “Limm” was utilized to screen differentially expressed immune-related genes (DE-IRGs) between the two immune score subgroups.

Next, MM samples were divided into a HL and high immune group (Immune.high (IMH)), a HH and IML and a mixed group according to the hypoxia and immune score levels. Simultaneously, the same procedures were applied to obtain DE-H-IRGs.

Finally, the DE-H-IRGs, DE-HRGs and DE-IRGs were overlapped to obtain two gene sets including risk DEGs and protective DEGs. A Venn diagram was utilized to visualize the results. GO and KEGG pathway enrichment analyses were employed to risk DEGs and protective DEGs by the DAVID (15).

### Gene signature identification and signature construction

The “Limma” was applied to select DEGs between MM and normal samples in the GSE47552 dataset (p< 0.05, |log2FC| > 0.5). The Benjamini & Hochberg method was applied for multiple test correction (16). The key DE-H-IRGs were developed by overlapping DEGs (tumor vs normal) with the risk DEGs and protective DEGs. DE-H-IRGs were further analyzed by univariate Cox analysis, and that satisfied with the p< 0.01 were regarded as prognosis DE-H-IRGs. LASSO analysis was used to select the most prognostic gene signature by the “glmnet”. The risk score of the model was calculated by the following formula (Y. 8).


$$\text{Risk score}=\sum_{i=1}^{n}{\text{Expr}}_{\text{gene}}\left(\text{i}\right)\;\times {\text{Coeff}}_{\text{gene}}\left(\text{i}\right)$$

### Assessment of the signature

MMs were grouped into the high- and low-risk group according to the median risk score. K-M analysis was utilized to analysis survival probability differences between the risk groups. The model was further verified by ROC and risk curves. GSE136337 dataset as an external validation cohort for model validation. Moreover, independent prognostic factors were selected from clinicopathological features (gender, age, albumin, b2m, ldh, iss) and risk score by Cox analysis (P< 0.05). Additionally, a nomogram was established for predicting the survival of MM in each independent prognostic factor by the “RMS” (version 6.2-0).

### Estimation of immune cell infiltration

To investigate the immune cells infiltrations in the risk groups, the CIBERSORT and LM22 gene set were employed to compute the proportion of 22 types of immune cells in all samples. Wilcoxon test was employed to screen for significantly different immune cells. Then, Spearman’s method was employed to calculate the relationship between each signature and these different immune cells.

### Functional analysis of DEGs

“ClusterProfiler” was used to analyze the biological process (BPs) and KEGG pathways of DEGs in both risk groups. In addition, the GSEA was utilized to find some specific pathways.

### HLA system and immune checkpoint analyses

To explore the difference between the HLA family and immune checkpoints in patients in the risk groups, the expression of the HLA family (17) and immune checkpoints were compared by Wilcoxon test, and the results were plotted by “GGplot2”.

### qRT-PCR validation

Total RNAs were extracted from biopsy and reverse transcribe into cDNA according to manufacture protocols(Toyobo, Japan). qPCR were performed by ABI 7500 applied system. All primers were available in **Table 1**. GAPDH and 2^-ΔΔCt^ method were utilized to normalize and calculate the relative mRNA expression.

**Table 1:** Primers for 8 candidate hypoxia-immune related genes and Gapdh.

GENE	Primer
DDIT4-F	CTCCTCTTCGCCCTCGTCCT
DDIT4-R	AGCCACTGTTGCTGCTGTCC
DNTT-F	CACATCGTAGCAGAGAACAA
DNTT-R	CTGACACGCATACTGGGAGA
FAM133A-F	CGTTCATACAAATCATCCCA
FAM133A-R	TTCTCGCTCTTCACACCTTC
CHRDL1-F	CCCCAGTGAACAATAAGGTGA
CHRDL1-R	AGTGAGAGCGGTGGTAAGAAT
MYB-F	GTTCCATACCCTGTAGCGTTA
MYB-R	GGTTCTGTGTCTGCTGTCCTT
PRR15-F	CCAACAGCAGAAAGAAAAGC
PRR15-R	TGGGGGTCACCAGGAAAGCC
QTRT1-F	ATGGTGTCGCTGGTGTCTCT
QTRT1-R	CGTCTCGCTTGGTCATCTCT
ZNF275-F	CTTTTGGGCGTTCCTGTT
ZNF275-R	GAGTCCCCGTGCTGTCTG
GAPDH-F	CCCATCACCATCTTCCAGG
GAPDH-R	CATCACGCCACAGTTTCCC

### Gene silencing

siRNA were transiently transfected into NCI-H929 and RPMI8226 by transfection reagent INTERFERin^®^ (Poly-Plus Corporation). Cells were harvested after 48h post transfection and subjected to further experiments. siRNA sequences were listed in **Table 2**.

**Table 2:** SiRNA sequence.

GENE	SPECIES	SENSE(5'-3')
DDIT4	Homo sapiens	5′-GCAAGAGCUGCCAUAGUGUTT-3′

### Construction of stable cell lines

The sh-DDIT4 was cloned into the pLKO.1-egfp-puro vector. The stably expressed sh-DDIT4 H929 cell line were selected by medium with Puromycin (Life Technologies). 3 weeks later, puromycin-resistant cells were generated for next experiments.

### Cell proliferation analysis

2.0×10^4^ cells were seeded into 96-well plates and transfected. After 48 h of culture under normoxic (pO2, 21%) and hypoxic (pO2, 1%) conditions, FBS free medium with WST-8 solution(Enhanced Cell Counting Kit-8, 1:10) was added and incubated for 2h to detect cell proliferation. The OD value was detected by a microplate reader.

### Apoptosis analysis

Cells were transfected with siRNA and cultured under normoxic (pO2, 21%) and hypoxic (pO2, 1%) conditions for 48 h. Then, cells were collected and centrifuged at 1000rpm for 5 min followed by media removal. After staining with annexin V/FITC and PI (BD Biosciences), cells were kept in the dark at room temperature for 15 min. Flow cytometry analyses were performed for detecting cell apoptosis *via* a FACSCalibur flow cytometer (BD Biosciences). FlowJo software was applied to analyze the data.

### Transwell assay

For migration and invasion assays, cell suspensions (3×10^5^ and 5×10^5^ cells, respectively, in 200 μl of medium containing 0.2% BSA) were seeded on 4 μm pore size Transwell membranes (Costar, Corning Incorporated, NY, USA), which were coated (migration assay) or not coated (invasion assay) with Matrigel (BD Biosciences, NJ, USA). Medium in the lower chambers contains 10% FBS was added. After incubation, cells on the insert membranes were fixed and stained with crystal violet for 30 min. Then, migrated or invade cells were imaged with microscope (Nikon, Tokyo, Japan). Cells migrating into lower chambers within the 24 h normoxia incubation period (pO2, 21%) and hypoxia incubation period (pO2, 1%) were counted using FACSCalibur flow cytometer (BD Biosciences).

### Animals and groups

Six-week-old male NOD/SCID mice were fed in pathogen-free facility. 5 × 10^6^ NCI-H929 cells stably transfected with shRNA scramble or shRNA DDIT4 were subcutaneously injecting into the right flanks of mice to generated xenograft tumor model. NOD/SCID mice were divided into control and shDDIT4 groups. According to experimental conditions, mice were divided into four groups: normoxia control (CTL), normoxia shDDIT4 (CTL+shDDIT4), intermittent hypoxia control (IH), and IH shDDIT4 (IH+shDDIT4). Hypoxia mouse models were generated by intermittent hypoxia exposure as previously described (8). The oxygen concentration in the IH group varied from 21% to 6%. Hypoxia (O_2_ concentration: 6%~8%) and reoxygenation (21%) were alternated by a program with a cycle time of 120 seconds for 5 consecutive weeks. The oxygen concentration in the CTL group was kept at 21%. Mice weight in each group was determined every week. Tumor volume (length × width^2^ × 0.5) was recorded every third day. Tumor tissues were harvested 35 days later. A K-M survival plot was applied to analyze the survival of mice. All experiment were approved by the Animal Care and Use Committee of West China Hospital, Sichuan University.

### Statistical analysis

All analyses were performed with R version 3.4.1 and related packages. The experimental data of this study were repeated for more than three replicates. For other comparisons in this study, Student’s *t*-test was used to detect differences between groups. The selection of statistical methods is described in the specific study methods. Differences were considered statistically significant at P less than 0.05 (*p< 0.05, **p< 0.01, ***p< 0.001, **** *p*< 0.0001). Differences between groups were analyzed by GraphPad Prism 8.

## Results

### Differential expression analysis of HRGs

**Figure 1:**
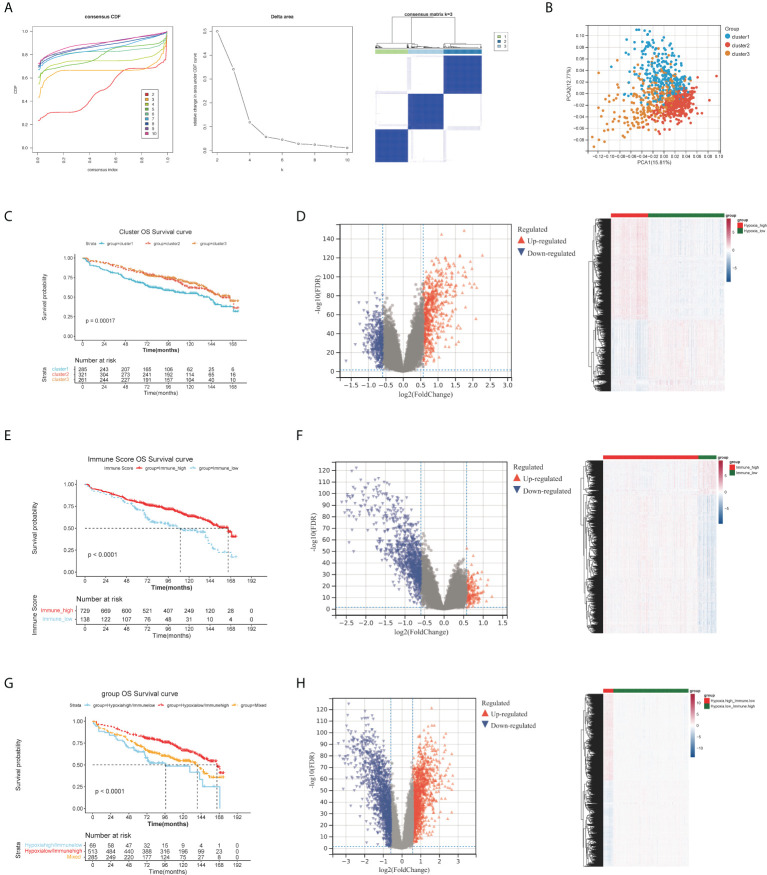
Differential expression analysis of HRGs, IRGs and H-IRGs. **(A)** The cumulative distribution function (CDF) value, the cluster diagram when K = 3. **(B)** Principal component analysis. **(C)** The K-M survival analysis among the three clusters. **(D)** Volcano plot and heatmap of DEGs between the HH and HL groups. **(E)** K-M analysis between the IMH and IML groups. **(F)** Volcano plot and heatmap of DEGs between the IMH and IML groups. **(G)** K-M analysis among the HL/IMH, HH/IML and mixed groups. **(H)** Volcano plot and heatmap of DEGs between the HL/IMH and HH/IML groups.

The 867 samples were grouped into three significantly different clusters based on the consensus clustering (**Figure 1A**). Principal component analysis (PCA) results illustrated the three clusters could be clearly distinguished (**Figure 1B**). By K-M analysis, we found the Cluster 1 patients exhibited a worse prognosis than patients of Clusters 2 and 3 (**Figure 1C**). Therefore, cluster 1 with the worst prognosis was defined as Hypoxia.high. Clusters 2 and 3 with better prognosis were combined and defined as Hypoxia.low. Finally, there were 582 and 285 MMs in the HL group and HH group respectively.

1407 DE-HRGs were screened, including 823 upregulated genes (risk DE-HRGs) and 584 downregulated genes (protective DE-HRGs) (**Figure 1D** and **Table S1**). Moreover we found that in Hypoxia.high group, *PRDM1*, *SPATS2*, *BMP6*, *PLPP5*, *EIF2AK3* and *TXNDC15* were significantly upregulated, and. *NFIX*, *CTBP2* and *SVBP* were significantly downregulated (**Figure 1D**).

### Differential expression analysis of IRGs

729 and 138 MMs were grouped into the IMH and IML groups based on the optimal immune score cutoff (2581.809). Moreover, K-M analysis illustrated that the overall survival (OS) time of MMs in the IMH group was longer (**Figure 1E**). We screened 1405 DE-IRGs between groups with high and low immune scores including 1129 upregulated genes (risk DE-IRGs) and 276 downregulated genes (protective DE-IRGs) (**Figure 1F** and **Table S2**). **Figure 1F** visualized the expression of DEGs in the two Immune groups. High levels of *MNDA*, *FCN1*, *GCA* and *PLBD1* were accumulated in the IMH group.

### Differential expression analysis of H-IRGs

A total of 867 MM samples were classified according to hypoxia status and immune score and were classified into HL/IMH (n = 513), HH/IMH (n = 69) and mixed groups (n = 285). K-M analysis result demonstrated that there was a significant difference in the prognosis of MMs among these three groups (p< 0.0001), and the prognosis of MMs in the HL/IMH group was better than others (**Figure 1G**). We screened 3474 DE-H-IRGs (HH/IML vs HL/IMH), consisting of 1743 upregulated genes (risk DE-H-IRGs) and 1731 downregulated genes (protective DE-H-IRGs) (**Figure 1H** and **Table S3**). In addition, the expression of DEGs in different groups were visualized in **Figure 1H**. High levels of *TNFRSF17*, *BMP6* and *MZB1* accumulated in the HL/IMH group.

### Screening and functional analysis of protective and risk genes

**Figure 2:**
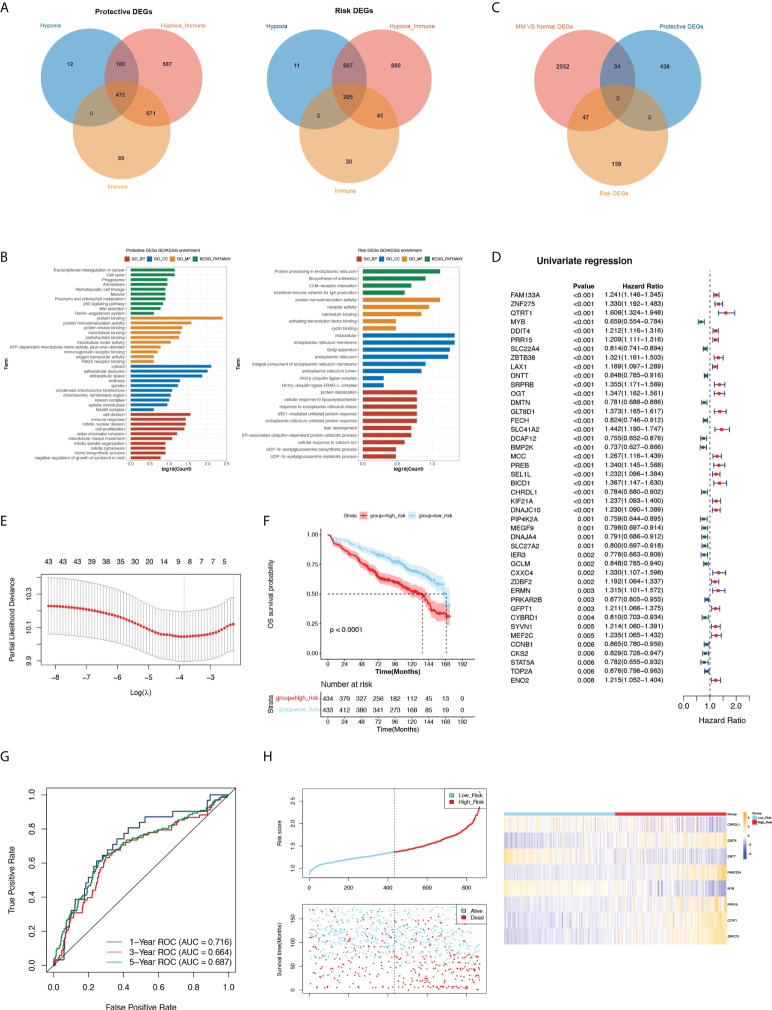
Analysis protective and risk genes, construct and assess the prognostic gene signature related to MM. **(A)** Venn diagram of protective and risk DEGs. **(B)** GO and KEGG terms enriched in protective and risk DEGs. **(C)** The Venn diagram among the protective DEGs, risk DEGs and DEGs related to MM. **(D)** Forest plot of hazard ratios for 44 prognostic DE-H-IRGs. **(E)** Threefold cross-validation for tuning parameter selection in the LASSO model. **(F)** K-M analysis between the high- and low-risk groups. **(G)** ROC curve at 1-, 3- and 5-years of prognostic value of the prognostic index. **(H)** The distributions of risk score, survival status and expression profile of signature genes between the risk groups.

To obtain more meaningful DEGs, we overlapped the three groups of risk DEGs and protective DEGs (**Figure 2A**). There were 472 protective DEGs and 205 risk DEGs with supporting evidence from all three groups.

The functional enrichment results revealed that protective DEGs were enriched in 191 GO terms and 13 KEGG pathways, including the immune response, innate immune response, neutrophil chemotaxis, and other immune-related pathways. The risk DEGs were enriched in 37 GO terms and 4 KEGG pathways, among which immune-related pathways were enriched such as adaptive immune response (**Figure 2B**).

### Construction of a prognostic gene signature related to MM

We screened 2633 DEGs between normal and MM patients in GSE47522, including 817 upregulated genes and 1816 downregulated genes. A Venn diagram showed the overlap of MM vs normal DEGs, protective DEGs and risk DEGs, and 81 key DE-H-IRGs overlapped (**Figure 2C**). The univariate Cox analysis was utilized on 81 key genes in the training set (GSE136324), and 44 prognostic DE-H-IRGs statistically related to the OS time of MMs were identified (**Figure 2D**). Then, an 8-gene signature (including *CHRDL1*, *DDIT4*, *DNTT*, *FAM133A*, *MYB*, *PRR15*, *QTRT1*, and *ZNF275*) was identified using the LASSO regression algorithm (**Figure 2E**).

### Assessment of the prognostic value of the 8-gene signature

434 and 433 MM patients were selected to high- and low-risk groups with the median risk score (1.368411315). It can be found that the OS of MMs in the low-risk group was longer in the GSE136324 (**Figure 2F**). The AUC of 1-year ROC curve in the training cohort was greater than 0.7, which indicated the good prognostic value of the signature (**Figure 2G**).

The risk curves of the signature demonstrated that the sample could be clearly classified into risk groups (**Figure 2H**). Analysis of the expression of the signature in the two risk groups showed that *CHRDL1*, *DNTT* and *MYB* genes expressed higher in high-risk MMs. The expressions of *DDIT4*, *FAM133A*, *PRR15*, *QTRT1* and *ZNF275* were higher in the low-risk group (**Figure 2H**). Additionally, the prognostic significance of this 8-gene signature was verified in GSE136337. (**Figure S1A-E**).

### The risk score is an independent prognostic indicator

**Figure 3:**
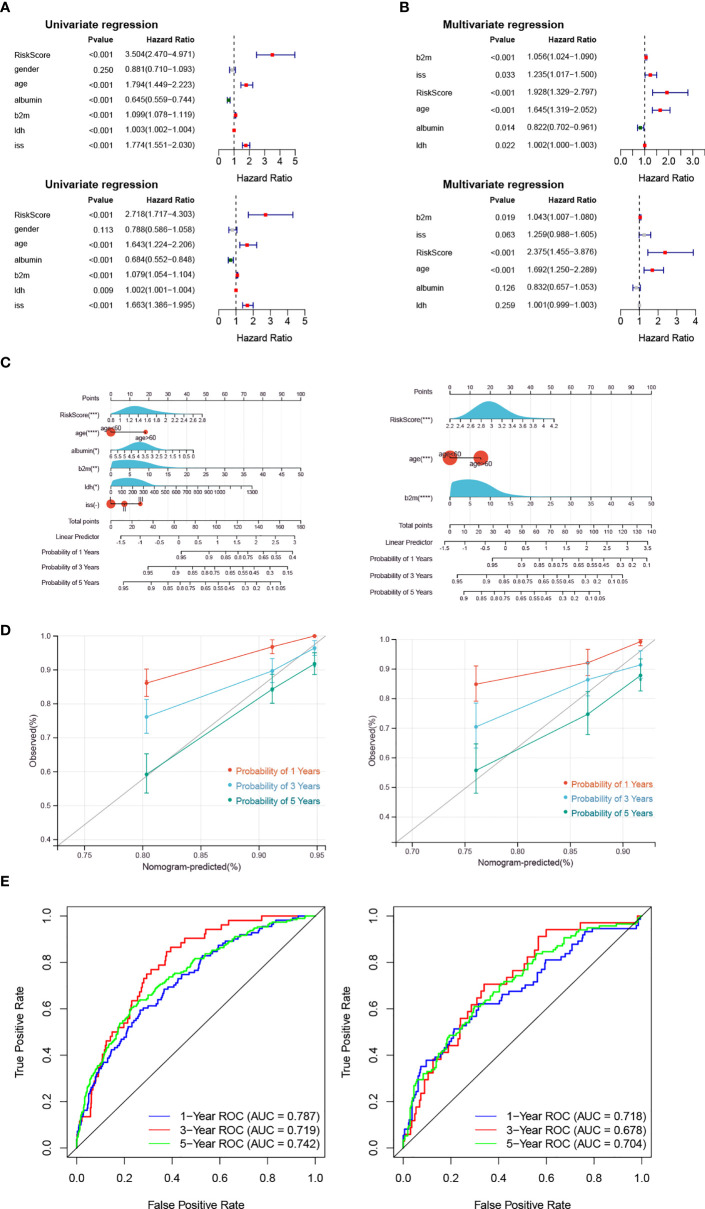
The Risk Score is an independent prognostic indicator. **(A, B)** The forest plot of hazard ratios for clinicopathological characteristics by Cox analysis. The upper panel shows the GSE136324 training cohort, and the lower panel shows the GSE136337 validation cohort. **(C–E)** The nomogram was used to show the survival probability at 1-, 3- and 5-years. The correction curves and the ROC curves were utilized to verify the efficiency of the nomogram. The left panel shows the GSE136324 training cohort, and the right panel shows the GSE136337 validation cohort.

Univariate Cox analysis utilized that risk score, age, albumin, b2m, ldh and iss were significantly related to patient OS (**Figure 3A**). Then, the six factors were applied for multivariate Cox analysis, and b2m, risk score and age were significantly related to OS in the training and validation cohorts, which could be considered independent risk factors for MM (**Figure 3B**).

Moreover, the risk score and clinical factors (age, albumin, b2m, idh, iss) were used to utilize a nomogram (**Figure 3C**). The survival probability at 1-, 3- and 5-years could be predicted according to the total score of the nomogram. The slope of the probability of 5-year was close to 1 in the correction curve, and the AUCs of 1-year and 5-year were greater than 0.7 in the ROC curve, which indicated that the nomogram could be used as an effective model (**Figures 3D, E**).

### Analysis of immune infiltration and immunotherapy responses in high- and low-risk groups

**Figure 4:**
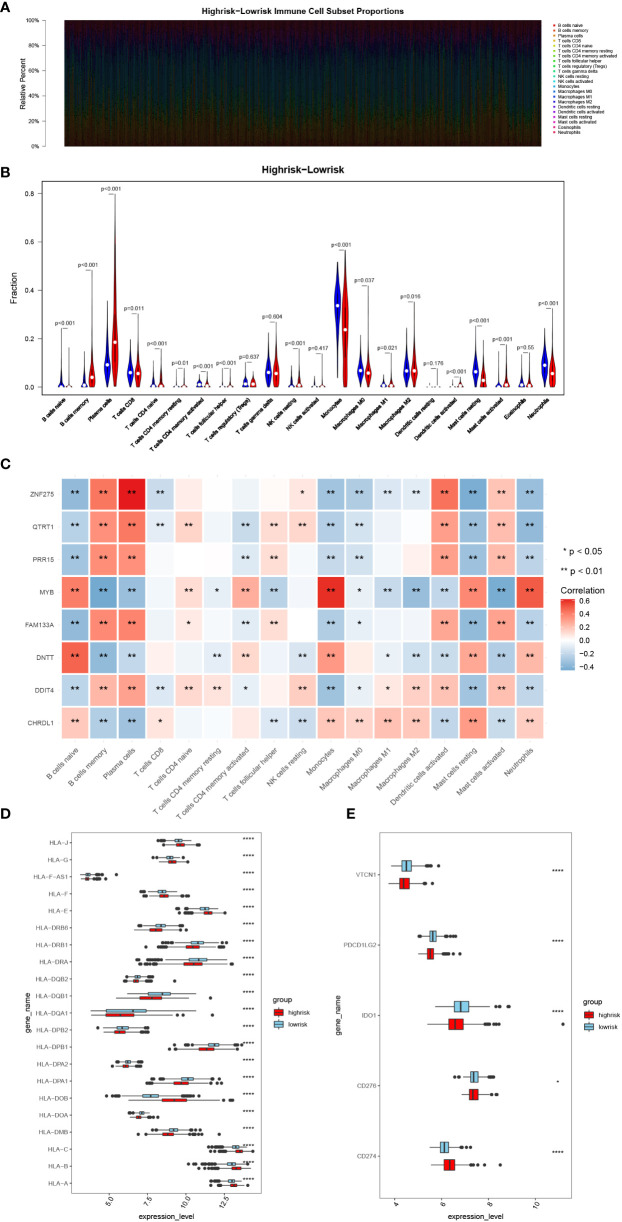
Analysis of immune infiltration and immunotherapy responses in high- and low-risk groups. **(A)** Heatmap of immune cell subset proportions. **(B)** Violin plot of the infiltration abundance of 22 immune cell types in two groups. **(C)** Heatmap of the correlation between signatures and differentiated immune cells. **(D, E)** The HLA family and immune checkpoint genes expression in the two groups. (*, p< 0.05; **, p< 0.01; ****, p< 0.0001; vs. CONTROL).

The CIBERSORT result demonstrate that among the MM samples in both risk groups, we found 17 types of immune cells were differentially accumulated (**Figure 4A, B**), such as B cells naïve and Plasma cells. Correlation analysis between each signature and the above differentiated immune cells showed that *ZNF275* was positively correlated with Plasma cells, and *MYB* was positively correlated with Monocytes and Neutrophils (**Figure 4C**). Furthermore, we found significant difference in the expressions of the HLAs and immune checkpoints between the risk groups (**Figure 4D, E**). The expressions of the immune checkpoints IDO1, VTCN1, PDCD1LG2 and CD274 were higher in the high-risk group.

### Differential expression analysis

**Figure 5:**
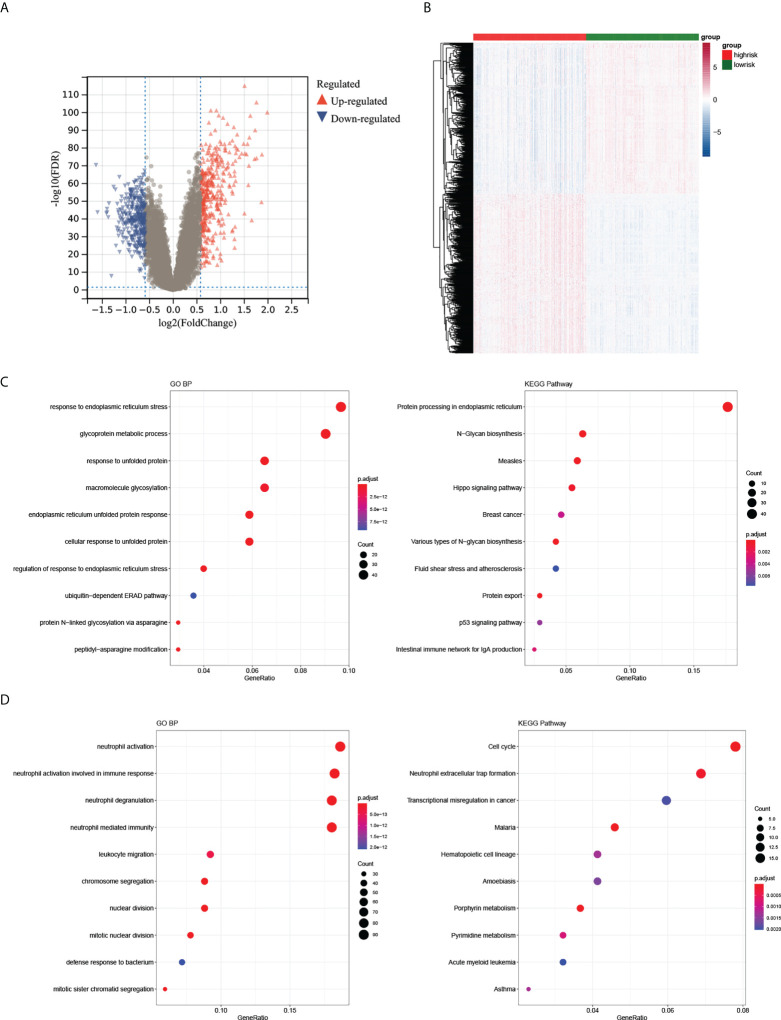
Differential expression analysis of high- and low-risk groups. **(A, B)** The volcano plot and heatmap of DEGs between the two groups. **(C, D)** The top 10 GO BP and KEGG terms of up- and downregulated DEGs in the two groups.

1092 DEGs (561 upregulated and 531 downregulated) were obtained (**Figure 5A**). The heatmap demonstrated the expression of DEGs in the high- and low-risk groups (**Figure 5B**). Moreover, we found that upregulated genes were enriched in 201 GO BP terms and 29 KEGG pathways including negative regulation of immune system processes and other immune-related pathways. Downregulated genes were enriched in 255 GO BP terms and 27 KEGG pathways such as neutrophil mediated immunity, regulation of adaptive immune response and other immune related pathways. Collectively, these DEGs were associated with autoimmunity (**Figures 5C, D**). The GSEA results demonstrated that the enriched biological processes of the high-risk group mostly involved responses to endoplasmic reticulum stress, glycosylation, cellular responses to topologically incorrect proteins and responses to topologically incorrect proteins. The enriched biological processes of the low-risk group were mainly involved in the response to acetylcholine, digestion, hydrogen peroxide catabolic processes and metal ion export. The KEGG pathways with the most abundant genes in the high-risk groups were N glycan biosynthesis, protein export, Vibrio cholerae infection, and the hedgehog signaling pathway. (The GSEA results were shown in **Supplementary Material 1**).

### Validation of the expression of the 8-gene signature

**Figure 6:**
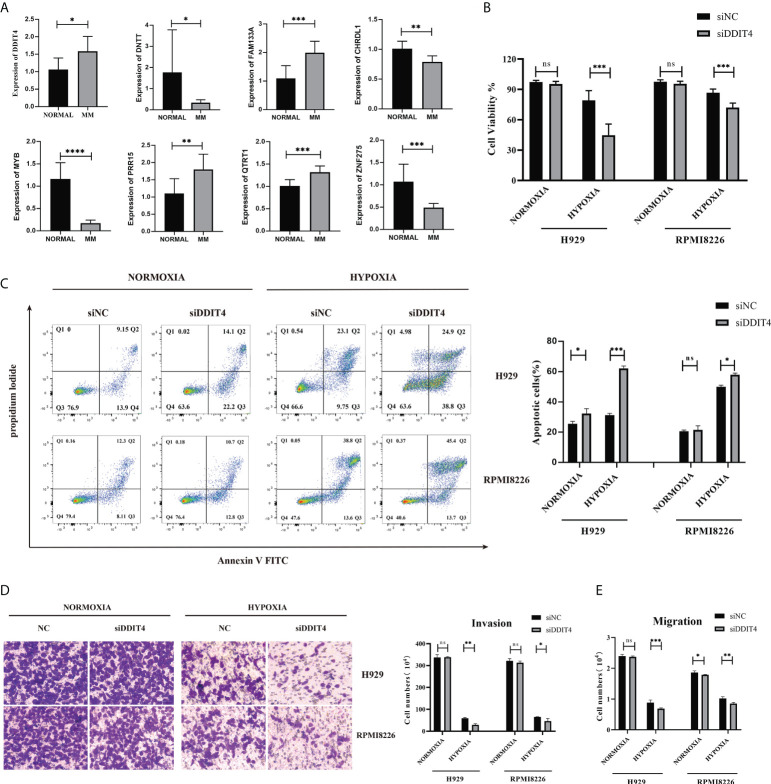
Validation of the expression of the 8-gene signature. The gene expression levels of *CHRDL1, DDIT4, DNTT, FAM133A, MYB, PRR15, QTRT1* and *ZNF275* between normal and bone marrow from 9 MM patients and peripheral blood samples from 7 control patients. **(A)** The relative expression levels of the 8 genes compared with *GAPDH* in normal (n = 7) and RRMM (n = 9) samples (*, p< 0.05; **, p< 0.01; ***, p< 0.001; ****, p< 0.0001). **(B)** The knockdown of DDIT4 reduced the cell viability of two myeloma cell lines relative. **(C)** The knockdown of DDIT4 increased the apoptosis rate of two myeloma cell lines. **(D, E)** Invasion and migration ratio of MM cells toward four groups through Transwell membranes (5-μm pore size) were assessed. Independent experiments were performed 3 times. n = 5 per group (**, p< 0.01; ***, p< 0.001; vs. CONTROL; ns, no significance).

The qRT–PCR results showed a significant upregulation in the expressions of *DDIT4*, *FAM133A*, *PRR15* and *QTRT1* in patients. Higher expression of *DNTT*, *CHRDL1*, *MYB* and *ZNF275* accumulated in the normal control (**Figure 6A**). Then, the function of the *DDIT4* was explored in RPMI8226 and NCI-H929. After knocking down the *DDIT4*, the cell viability was significantly inhibited in the hypoxia groups (**Figure 6B**). Correspondingly, we discovered that DDIT4 knockdown promoted cell apoptosis (**Figure 6C**) and impaired tumor migratory and invasive potential (**Figures 6D, E**).

### *DDIT4* inhibited tumor formation *in vivo* under hypoxia

**Figure 7:**
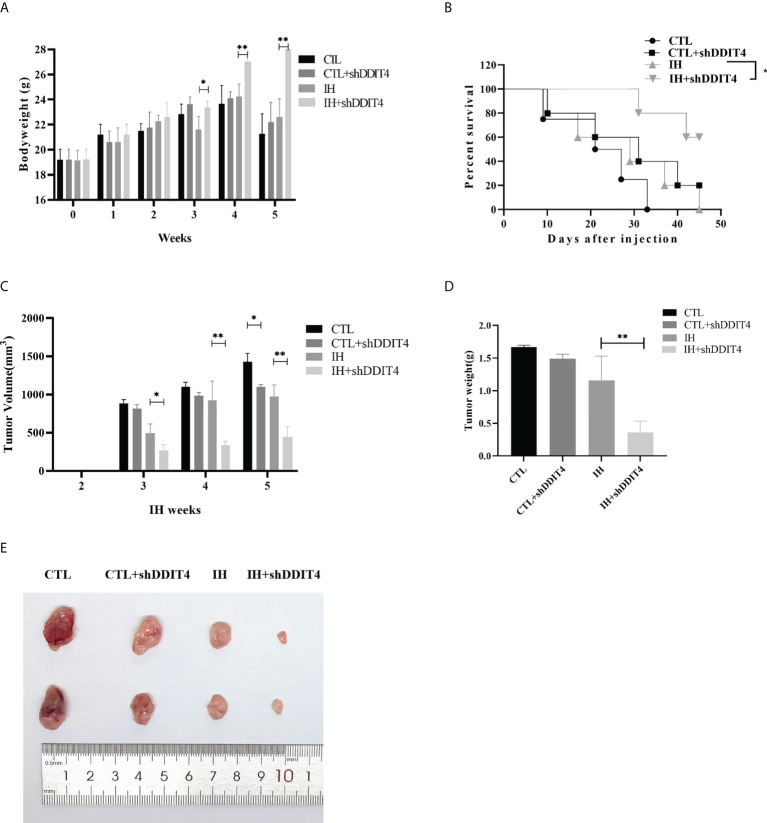
*DDIT4* restrained tumor formation *in vivo* under hypoxia. **(A)** The body weight of mice was measured after intermittent hypoxia (IH) administration in the CTL, CTL+shDDIT4, IH, and IH+shDDIT4 groups at 0~5 weeks. **(B)** The survival rate in different groups were detected for 5 weeks after injecting NCI-H929 cells into the mice. **(C, D)** Tumor volume and weight were measured in the 5th week after intermittent hypoxia administration. All experiments were performed triplicate. **(E)** The representative tumor size was photographed in four groups. CTL, normoxia control; CTL+shDDIT4, normoxia shDDIT4; IH, intermittent hypoxia control; IH+shDDIT4, intermittent hypoxia+shDDIT4. Independent experiments were performed 3 times. n = 5 per group (*, p< 0.05; **, p< 0.01; vs. CONTROL).

We constructed a murine xenograft model to confirm the effect of *DDIT4* in MM. Mice in the IH group lost body weight clearly at weeks 3 - 5 compared to the IH+shDDIT4 group (**Figure 7A**). Meanwhile, the K-M curve revealed that the survival rate of the IH+shDDIT4 group was obviously higher (**Figure 7B**). Moreover, in the IH+shDDIT4 group, both tumor volume and tumor weight were significantly reduced at 3 weeks under hypoxia (**Figures 7C-E**).

## Discussion

Multiple myeloma displays characteristics of plasma cells malignant proliferation. Hypoxia and the immune microenvironment play vital roles in the carcinogenicity and evolution of MM (18, 19). A recent study showed that a gene marker (*SKY92*) that effectively predicted treatment response and prognosis in MM by combining with the International Staging System (20). However, researchers have found that many previous genetic signatures do not appear to have overlapping genes (21). Therefore, novel therapeutic targets and disease prognostic indicators needs to be explored for next treatment of MM patients.

In this study, we overlapped the three groups of DEGs to estimate risk DEGs and protective DEGs, and we explored their potential functions in MM through functional enrichment analysis. There was an association between protective DEGs and immunity, including the immune response, innate immune response, neutrophil chemotaxis, and positive regulation of T-cell proliferation. In the innate immune response to tissue injury or infection, neutrophils are recruited to the inflammatory site. Neutrophil chemotaxis inhibitors were found in the sera of myeloma patients (22). In immunotherapy against MM, positive regulation of T-cell proliferation played a crucial role. In patients with early-stage MM related to advanced diseases, clonal CD8+ T-cell expansions were significantly more frequent (23). It has become evident that endogenous T cells can be used as a treatment for multiple myeloma (24).

The tumor microenvironment (TME) make up tumor tissue and their clinicopathological significance in predicting curative effects and therapeutic effects in MM (25). Scharping et al. (26) found that metabolic pressure originating from mitochondria under hypoxia could accelerate T-cell dysfunction and failure (Da Vià et al.). We found that 17 kinds of immune cells differ between the low- and high-risk groups by evaluating the proportion of immune cells (**Figure 4B**). Activated memory CD4^+^ T cells were significantly higher in the low-risk groups. Several studies revealed that adoptive transfer of idiotype-specific CD4^+^ T cells might play the role of resisting MM (27, 28).We hypothesized whether these memory cells are adoptively transferred to idiotype-specific CD4^+^ T cells. Single-cell sequencing analysis was applied to further clarify the origin and characteristics of these activated memory CD4^+^ T cells from low-risk groups.

We identified that hypoxia-immune-related prognostic DEGs, in MM patients, statistically correlated with the overall survival by K-M survival analysis. In the high-risk group, *ZNF275*, *FAM133A*, *PRR15*, *QTRT1* and *DDIT4* were significantly higher than the low-risk group. Foltyn et al. (29) found that knockout of the *DDIT4* could make tumor cells sensitive to hypoxia, while overexpression of *DDIT4* could enhance cell proliferation and promote resistance to temozolomide, radiotherapy and hypoxia. Similarly, we found that knockdown of the *DDIT4* inhibited MM cell viability, migration and invasion potential and promoted cell apoptosis under hypoxia. We also demonstrated that *DDIT4* inhibited tumor formation in a xenograft tumor mouse model under hypoxia. Now, the role of *DDIT4* in MM has not been elucidated. It can be regarded as a potential target for anti-MM therapy or as a molecular marker of the hypoxic state in our research.

Human leukocyte antigens (HLAs) are composed of the major histocompatibility complexes that recognized self and non-self antigens. Christopher MJ et al. (30) found that the lower expression of HLA-DPA1 was associated with immune function-related pathways dysregulation, which caused acute myeloid leukemia relapse after transplantation. We found that *HLA-DPA1* expression was lower in the high-risk groups. Notably, a recent study considered that downregulated *HLA-DPA1* expression was related with poor outcome in MM (31), which was consistent with our research. Known as surface antigen differentiation cluster 274 (*CD274*), programmed cell death ligand 1 (*PD-L1*) located in the cell membrane and endometrial system. *PD-L1* was detected on hematopoietic and nonhematopoietic healthy tissue cells (32). The mRNA level of *PD-L1* was higher in MM and RRMM patients than in healthy controls (33). We also found that expression of CD274 in the high-risk groups was enhanced. Considering the poor effect of *PD-L1* treatment alone, it is necessary to clarify the response of high-risk patients to *PD-L1* treatment and its mechanism in forward study.

In the study, the prognosis and stratification of MM were evaluated by transcription and microenvironmental remodeling. Our study illustrated that the survival of patients with MM was linked to the hypoxia remodeled tumor microenvironment and relative immune status. Hypoxia and immune status were found to be significantly related to prognosis by stratifying patients according to clinicopathological risk factors. Univariate regression and multivariate regression were applied to analyze independent prognosis and the risk score of each clinical factor. Finally, we screened an eight-gene signature based on hypoxia-immunity as a prognostic classifier and verified its efficiency in risk stratification. For our study, some limitations must be acknowledged. First and foremost, this is a retrospective study, so the prognostic robustness and clinical usefulness of hypoxia-immune related gene signatures need further examination. Second, we verified external datasets, the relevant regions information of patients involved in this study was not analyzed *in vitro* or in patient samples. Therefore, our findings will be further verified by multicenter RCT studies. Meanwhile, due to limited biology investigation, more experiments in cell and animal model will be performed to elucidate how the gene signatures modulate the outcome of MM.

## Data availability statement

The original contributions presented in the study are included in the article/**Supplementary Material**. Further inquiries can be directed to the corresponding author.

## Ethics statement

The studies involving human participants were reviewed and approved by the ethics committee of West China Hospital, Sichuan University. Written informed consent for participation was not required for this study in accordance with the national legislation and the institutional requirements. The animal study was reviewed and approved by the Animal Care and Use Committee of West China Hospital of Sichuan University.

## Author contributions

ZY and BQ conceive and design research, obtain original research data, analyze and interpret results. LL, HZ, and JX recruited patients. ZY analyzed the data and wrote the manuscript. ZY and TN revised the manuscript and interpreted the results. All authors discussed and provided critical comments and approved the final version for publication.

## Funding

This work was funded by support from the Post-Doctor Research Project, West China Hospital, Sichuan University (No. 2021HXBH085), Incubation Program for Clinical Trials (No.4619HXFH030), Achievement Transformation Project (No. CGZH21001), 1.3.5 Project for Disciplines of Excellence, West China Hospital, Sichuan University (No. ZYJC21007), and Translational Research Grant of NCRCH (No. 2021WWB03).

## Acknowledgments

We thank the participated patients. We also thank Dr. Yang Yang for technical supports.

## Conflict of interest

The authors declare that the research was conducted in the absence of any commercial or financial relationships that could be construed as a potential conflict of interest.

## Publisher’s note

All claims expressed in this article are solely those of the authors and do not necessarily represent those of their affiliated organizations, or those of the publisher, the editors and the reviewers. Any product that may be evaluated in this article, or claim that may be made by its manufacturer, is not guaranteed or endorsed by the publisher.

## Supplementary material

Supplementary Figure 1 GSE136337 validation cohort. **(A)** The K-M survival analysis between the high- and low-risk groups. **(B)** The ROC curve at 1, 3 and 5 year of prognostic value of the prognostic index. **(C–E)** Risk score, survival time and distribution of signature genes expression pattern between the two groups.
