# Supplementary figures and images for: An emerging prognosis prediction model for multiple myeloma: Hypoxia-immune related microenvironmental gene signature
Source: Front Oncol. 2022 Aug 30;12:992387. doi: 10.3389/fonc.2022.992387 (PMC9468480; doi:10.3389/fonc.2022.992387)

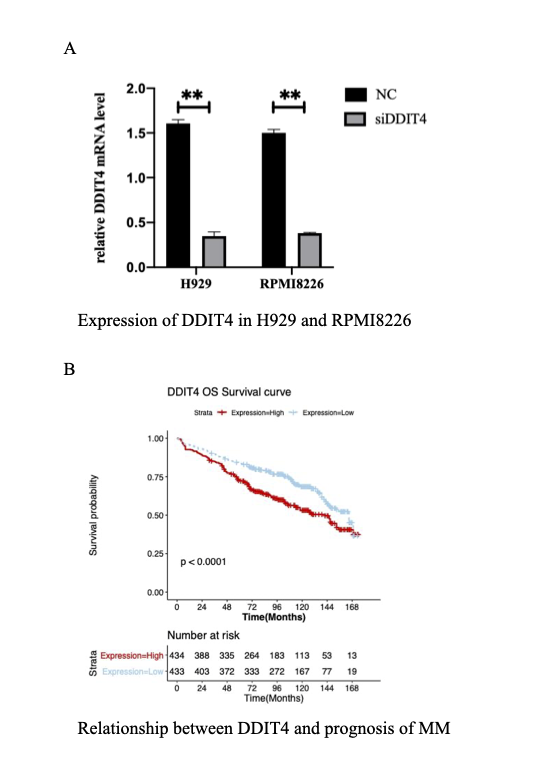

Supplement: Supplementary Figure 1 — GSE136337 validation cohort. (A) The K-M survival analysis between the high- and low-risk groups. (B) The ROC curve at 1, 3 and 5 year of prognostic value of the prognostic index. (C–E) Risk score, survival time and distribution of signature genes expression pattern between the two groups. [file Image_1.tiff]
